# Re: Percutaneous tibial nerve stimulation versus electrical stimulation with pelvic floor muscle training for overactive bladder syndrome in women: results of a randomized controlled study

**DOI:** 10.1590/S1677-5538.IBJU.2022.0168

**Published:** 2022-05-02

**Authors:** Necmettin Yildiz

**Affiliations:** 1 Department of Physical Medicine and Rehabilitation Pamukkale University Faculty of Medicine Denizli Turkey Department of Physical Medicine and Rehabilitation, Pamukkale University Faculty of Medicine, Denizli, Turkey

*To the editor,*

I thank the authors of “Percutaneous tibial nerve stimulation versus electrical stimulation with pelvic floor muscle training for overactive bladder syndrome in women: results of a randomized controlled study ( 1 ).” I support the importance you attribute to intravaginal electrical stimulation (IVES) and percutaneous tibial nerve stimulation (PTNS) in the management of idiopathic overactive bladder (OAB). The authors stated that PTNS is more effective than IVES in women with idiopathic OAB. Women with antimuscarinic naive OAB were included in this study ( 1 ). However, it is known that many patients with idiopathic OAB receive pharmacological treatment before reaching a conservative treatment option such as IVES. As the authors stated, in common practice, antimuscarinic agents are frequently used as an initial treatment although burdened by a low adherence, and these patients need protracted treatment with periodic controls.

## First-line or third-line?

What is the ranking of IVES and PTNS among the treatment options in patients with idiopathic OAB? Some authors listed the treatment options in idiopathic OAB as follows; first-line - behavioral therapy (lifestyle modifications, pelvic floor muscle training, bladder training, timed voiding), second-line - pharmacologic (antimuscarinic, beta-3 agonists), and third-line - neuromodulation/chemodenervation (PTNS, sacral neuromodulation, intradetrusor botulinum toxin). IVES is involved in pelvic floor muscle training as a first-line treatment option ( 2 ). On the contrary, some authors stated that “the first-line treatment of idiopathic OAB includes behavior modification and physical therapy, and neuromodulation methods are used as third-line therapy in cases refractory to first-line and second-line (pharmacological) treatment. IVES, PTNS, and sacral neuromodulation are included as neuromodulation options” ( 3 , 4 ). Furthermore, as you know, some studies included subjects not using antimuscarinics within the last 4 weeks or antimuscarinic-naive patients with OAB ( 1 , 5 ), while some included patients with OAB who were unresponsive or intolerant to antimuscarinics ( 3 ).

As a result, IVES and PTNS appear to be effective therapies used both as first-line treatment, as well as in managing refractory patients with idiopathic OAB. This needs to be expressed more clearly. Would it be more effective on the first line or the third line? or in other words; is there a difference in response to IVES and PTNS in antimuscarinic naive and refractory patients with OAB? Clarification of the ranking of both PTNS and IVES among treatment options in patients with OAB will be possible with the increase of qualified studies addressing this issue. This would make it easier to understand the rankings of IVES and PTNS among treatment options in patients with idiopathic OAB.

The Author
